# A1 USING HUMAN NEONATAL ORGANOIDS TO EXPLORE GUT-IMMUNE SYSTEM INTERACTIONS OF THE NEONATAL INTESTINE

**DOI:** 10.1093/jcag/gwab049.000

**Published:** 2022-02-21

**Authors:** J M Allaire, Z Sharafianardakani, B Poon, H Piper, K Jacobson, P Lavoie, B Vallance

**Affiliations:** 1 Pediatrics, University of British Columbia, Vancouver, BC, Canada; 2 BC Children’s Hospital, Vancouver, BC, Canada

## Abstract

**Background:**

Necrotizing Enterocolitis (NEC) affects around 10% of preterm babies and is one of the leading causes of death for newborns. NEC is characterized by exaggerated inflammation of the intestinal mucosa, possibly triggered by aberrant exposure to gut microbes, leading to hypoxic conditions and the death of intestinal tissues. It has been hypothesized that NEC develops when the immature intestine (epithelium and immune system) is unable to properly balance these new microbial interactions. To date, NEC is poorly understood and due to the difficulty of modeling the human neonatal intestine, few therapeutic options are available.

Intestinal epithelial cells (IEC) are important players in promoting beneficial host-microbe interactions in the gut, being the primary barrier that separates the host’s mucosal immune system from luminal microbiota, as well as key players in mediating signaling between microbes and the host. Based on their location, IEC are also subject to injury associated with maladaptive immune responses against gut microbes. Many studies have shown that immune cells (such as T helper 17 cells) interact with IEC to promote gut health and function. These interactions include educating IEC on how to respond to, and fight pathogenic microbes, yet also remain tolerant to commensal microbes.

**Aims:**

This project seeks to develop an *in vitro* human neonatal intestinal organoid model to study developmental changes in IEC and their functional interactions with neonatal Th17 cells.

**Methods:**

3D organoids were established from human neonatal intestinal biopsies and then co-cultured with the supernatant of differentiated Th17 cells or with recombinant cytokines, IL-17 and IL-22. Changes in barrier function, cell proliferation, production of mucins and anti-microbial peptides (AMP) were analyzed by qPCR and immunostaining.

**Results:**

Using 3D neonatal organoids we observed that the supernatants from neonatal Th17 cells (containing IL-17, IL-22 etc.) promoted the proliferation, differentiation and barrier function of the neonatal epithelium. By using specific recombinant cytokine (IL22, IL17) and neutralizing IL-22 antibodies in parallel, we demonstrated that the high levels of IL-22 produced by neonatal Th17 cells specifically induced proliferation of IEC, AMP and mucus production as compared to control media treated organoids, as shown by increases in Ki67, Reg3γ and Muc2 markers.

**Conclusions:**

This experimental model mimicking the neonatal intestinal environment can be used to study interactions between neonatal IEC and immune cells. Our findings can provide clinically relevant information and clues to how developmental changes in the newborn intestine can influence susceptibility to NEC while demonstrating our development of a simple, yet accurate and clinically applicable model of the neonatal gut.

**Funding Agencies:**

CAG, CIHRBCCHRI, CCC, C.H.I.L.D. Fdn

